# A double-blinded, randomized controlled clinical trial of hydrogen inhalation therapy for idiopathic sudden sensorineural hearing loss

**DOI:** 10.3389/fnins.2022.1024634

**Published:** 2022-11-24

**Authors:** Masahiro Okada, Hideo Ogawa, Taro Takagi, Eriko Nishihara, Tadashi Yoshida, Jun Hyodo, Yusuke Shinomori, Nobumitsu Honda, Takashi Fujiwara, Masato Teraoka, Hiroyuki Yamada, Shin-ichi Hirano, Naohito Hato

**Affiliations:** ^1^Department of Otolaryngology, Head and Neck Surgery, Ehime University Graduate School of Medicine, Toon, Japan; ^2^Department of Otolaryngology, Head and Neck Surgery, Ehime Prefectural Central Hospital, Matsuyama, Japan; ^3^Department of Otolaryngology, Ehime Prefectural Niihama Hospital, Niihama, Japan; ^4^Department of Otolaryngology, Head and Neck Surgery, Uwajima City Hospital, Uwajima, Japan; ^5^Department of Otolaryngology, Takanoko Hospital, Matsuyama, Japan; ^6^Department of Otolaryngology, Matsuyama Red Cross Hospital, Matsuyama, Japan; ^7^Department of Public Health Research, Kurashiki Clinical Research Institute, Kurashiki, Japan; ^8^Department of Research and Development, MiZ Company Limited, Kamakura, Japan

**Keywords:** sudden deafness, hydrogen, inner ear, free radical, hearing loss

## Abstract

**Background:**

Hydrogen (H_2_) has been reported to be effective in reducing hearing loss due to several causes in animal studies. However, no study has examined the effectiveness of H_2_ in treating hearing loss in humans. Thus, we investigated whether H_2_ is effective for the treatment of idiopathic sudden sensorineural hearing loss (ISSNHL).

**Materials and methods:**

We conducted a double-blind randomized controlled trial at six hospitals between June 2019 and March 2022. The study protocol and trial registration have been published in the Japan Registry of Clinical Trials (jRCT, No. jRCTs06119004). We randomly assigned patients with ISSNHL to receive either H_2_ (H_2_ group) or air as a placebo (control group) through inhalation combined with the administration of systemic glucocorticoids and prostaglandin E1. The primary outcome was the hearing threshold and changes in hearing threshold 3 months after therapy. In contrast, the secondary outcomes included the proportion of patients with a good prognosis (complete recovery or marked improvement).

**Results:**

Sixty-five patients with ISSNHL (31 and 34 in the H_2_ and control groups, respectively) were included in this study. The hearing threshold 3 months after treatment was not significantly different between the groups; 39.0 decibels (dB) (95% confidence interval [CI]: 28.7–49.3) and 49.5 dB (95% CI: 40.3–58.7) in the H_2_ and control groups, respectively. In contrast, the changes in hearing threshold 3 months after treatment was 32.7 dB (95% CI: 24.2–41.3) and 24.2 dB (95% CI: 18.1–30.3) in the H_2_ and control groups, respectively. Consequently, the changes in hearing threshold were significantly better in the H_2_ group than in the control group (*P* = 0.048). However, no adverse effects due to the inhalation of H_2_ gas have been reported.

**Conclusion:**

This is the first study to investigate the efficacy of H_2_ for the treatment of ISSNHL in humans. The results suggest that H_2_ therapy may be effective for ISSNHL treatment.

**Clinical trial registration:**

[https://jrct.niph.go.jp/re/reports/detail/10442], identifier [jRCTs06119004].

## Introduction

Idiopathic sudden sensorineural hearing loss (ISSNHL) is a sensorineural hearing dysfunction of unknown etiology characterized by a sudden onset and mostly unilateral hearing loss. A recent epidemiological survey in Japan showed that the incidence of ISSNHL occurs in 60.9 per 100,000 of the population (Nakashima et al., 2014). The pathogenesis of ISSNHL is still unknown; however, administration of corticosteroids, hyperbaric oxygen therapy, vasodilators, and intratympanic steroid injections are often used to treat ISSNHL (Kitoh et al., 2017; Chandrasekhar et al., 2019; Herrera et al., 2019). Although these treatments have been proposed to constitute the possible intracochlear mechanisms of the disease, the prognosis of ISSNHL remains insufficient (Kitoh et al., 2017).

Molecular hydrogen (H_2_) is an antioxidative agent that can prevent oxidative stress-induced diseases, such as brain infarction (Ono et al., 2017), myocardial infarction (Katsumata et al., 2017), post-cardiac arrest syndrome (Tamura et al., 2020), respiratory function in post-COVID-19 patients (Botek et al., 2022), and rheumatoid arthritis (Ishibashi et al., 2014), in human clinical trials. In addition, H_2_ has been reported to be effective in reducing hearing loss due to transient cochlear ischemia (Ogawa et al., 2018), loud sound (Lin et al., 2011; Zhou et al., 2012; Chen et al., 2014; Kurioka et al., 2014; Fransson et al., 2021), and ototoxic agents (Qu et al., 2012; Kikkawa et al., 2014; Fransson et al., 2017). Ogawa et al. (2018) reported that H_2_ was effective for hearing loss induced by cochlear ischemia in an animal model, which is thought to be the main cause of ISSNHL.

In previous animal studies, transient cochlear ischemia induced hearing loss via the depletion of the adenosine triphosphate (ATP) supply, glutamate excitotoxicity, and cell damage by the generation of free radicals (Hakuba et al., 1997, 2000; Maetani et al., 2003; Morizane et al., 2005). As H_2_ can selectively scavenge peroxynitrite and hydroxyl radicals produced after transient ischemia (Ohsawa et al., 2007), it has potential effectiveness for ISSNHL treatment. However, the effectiveness of H_2_ in sensorineural hearing loss has not yet been reported in humans.

In previous clinical studies using H_2_ inhalation, 1.3–3% of H_2_ was used for various diseases (Ishibashi et al., 2014; Katsumata et al., 2017; Ono et al., 2017; Tamura et al., 2020; Botek et al., 2022). A randomized controlled study reported that 3% H_2_ gas (1 h twice a day) was effective in patients with acute cerebral infarction (Ono et al., 2017). In addition, no adverse effects due to H_2_ gas have been reported in the previous studies (Ishibashi et al., 2014; Katsumata et al., 2017; Ono et al., 2017; Tamura et al., 2020; Botek et al., 2022) which is in contrast to the conventional treatments, including glucocorticoids, which have side effects. Our hypothesis is that H_2_ gas inhalation is a safe and effective treatment for ISSNHL. Thus, we investigated the effects of 3% H_2_ gas inhalation in patients with ISSNHL.

## Materials and methods

### Study design

This multicenter, double-blind, randomized clinical trial was approved by the Certified Review Board of Ehime University (No. 19EC002) and was performed from June 2019 through March 2022 at six hospitals in Ehime Prefecture, Japan. The study protocol and trial registration have been published in the Japan Registry of Clinical Trials (jRCT, No. jRCTs06119004;^1^ as it is written in only Japanese, details in this website are mentioned below). Furthermore, written informed consent was obtained from the patients.

### Study population

Patients who were diagnosed with ISSNHL at six hospitals in Japan (Ehime University Hospital, Ehime Prefectural Central Hospital, Ehime Prefectural Niihama Hospital, Uwajima City Hospital, Takanoko Hospital, and Matsuyama Red Cross Hospital), aged ≥ 18 years, and visited the hospital within 14 days of onset were included in this study. ISSNHL was defined as a unilateral sensorineural hearing loss of approximately 30 decibels (dB) over at least three test frequencies that developed within 3 days. All patients underwent magnetic resonance imaging and distortion products of otoacoustic emissions to prevent retrocochlear pathologies or functional hearing loss. Patients who were diagnosed with other diseases, including vestibular schwannoma, Meniere’s disease, or pelilymphatic fistula, were excluded from this study. Patients whose data were incomplete were also excluded from this study.

### Study intervention

Patients were randomly assigned (1:1) to receive either H_2_ by inhalation (H_2_ group) or placebo (inhalation of air, control group). Randomization was performed with stratification based on the hearing threshold (≥60 dB vs. <60 dB) and age (≥65 vs. <65 years).

All patients were treated with systemic glucocorticoid and prostaglandin E1 (PGE1). Prednisolone in doses of 60, 40, and 20 mg was administered sequentially at intervals of 3 days, respectively, while intravenous PGE1 was administered for 6 days. However, when the hearing was insufficiently recovered after the systemic administration of glucocorticoids, intratympanic injection of dexamethasone (thrice weekly) was performed if requested. In addition, H_2_ gas was inhaled through a cannula attached to the nose for 1 h twice daily for 6 days. Particularly, H_2_ gas for the inhalation was generated by electrolysis of water using a hydrogen gas supply apparatus (MHG-2000α, MiZ Company Limited, Kamakura, Japan), which comprises an electrolysis chamber, membrane, and electrode plates. The H_2_ gas was generated from the cathode surface and adjusted to a volume of 2 L per minutes to maintain the H_2_ gas concentration at 3%. In contrast, the air was inhaled using the placebo equipment in the control group instead of H_2_ gas.

The data, including age, complications, affected side, days from onset to treatment, and hearing threshold, were collected at each hospital by investigators.

### Randomization

Investigators at each hospital registered patients to the Clinical Therapeutic Trial Center at Ehime University Hospital using fax. Research coordinators at the center then generated a unique number and randomized the patient to group “A” or “B.” The randomization schedule was stratified by the hearing threshold (≥60 dB vs. <60 dB) and age (≥65 years vs. <65 years) in blocks of four, using Research Randomizer.^2^ The randomization schedule was blinded to the investigators and patients. The coordinator faxed the allocated group to the researcher. Patients were treated with gas supply apparatus labeled “A” or “B.” This labeling was performed by MiZ company Limited. Study investigators, research coordinators, patients, and nursing staff were blinded to this labeling.

### Study outcomes

The hearing outcomes were estimated 1 and 3 months after the treatment using a pure tone audiogram (PTA). This study defined severe and mild as the initial hearing threshold (a mean of 250, 500, 1 k, 2 k, and 4 k Hz thresholds) ≥60 dB and <60 dB, respectively. The primary outcomes in this study were the hearing threshold and its changes from onset to 3 months after treatment. Contrarily, the secondary outcomes were as follows: (1) hearing threshold and its changes from onset to 1 month after treatment, (2) ratio of good hearing prognosis (complete recovery and marked improvement), (3) hearing outcomes based on the severity of hearing threshold at the initial visit (<60 dB vs. ≥60 dB), (4) hearing outcomes, which depend on complications including hypertension, hyperlipidemia, and diabetes, and (5) side effects and safety of inhaling H_2_ gas. We used the hearing improvement criteria for ISSNHL defined by the Ministry of Health and Welfare in Japan (Kitoh et al., 2017).

### Statistical analysis

The data are expressed as mean and a 95% confidence interval (CI). The distribution normality was assessed using the Shapiro–Wilk test. The differences in the hearing outcomes between the H_2_ and control groups were analyzed using a one-tailed *t*-test or Fisher’s exact test to investigate the additional effects of H_2_ gas inhalation. Other parameters between groups were analyzed using a two-tailed *t*-test or Fisher’s exact test. In addition, multiple linear regression was used to predict hearing outcomes at each frequency based on age and treatment (H_2_ or placebo). All analyses were performed using JMP for Macintosh (SAS Institute Inc., Cary, NC, USA). The statistical tests were based on a significance level of *P* < 0.05.

## Results

**FIGURE 1:**
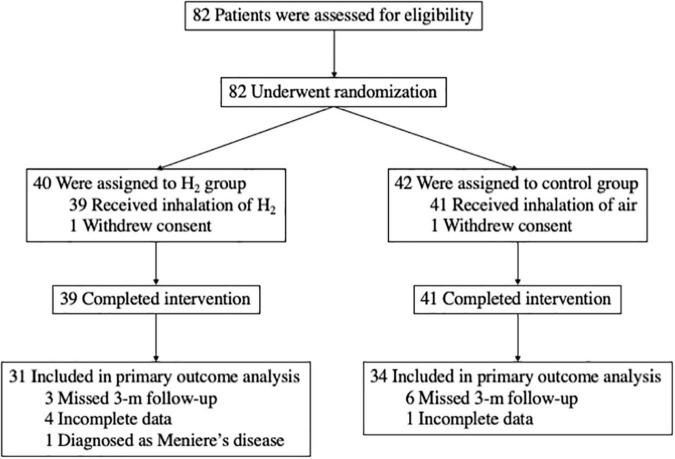
Study overview.

Overall, 82 patients consented to participate in the study (Figure 1) and were randomly assigned to either the H_2_ or control groups between June 2019 and December 2020. One patient in the H_2_ group and one in the control group withdrew consent, while 39 and 41 patients in the H_2_ and control groups, respectively, completed the intervention. In addition, eight patients in the H_2_ group were excluded from the analysis due to the missed 3-month follow-up (*n* = 3), incomplete data (*n* = 4), and diagnosis of Meniere’s disease (*n* = 1). In contrast, seven patients in the control group were also excluded because of the missed 3-month follow-up (*n* = 6) and incomplete data (*n* = 1). Ultimately, 65 patients (31 and 34 in the H_2_ and control groups, respectively) were included in the analysis.

Table 1 shows the baseline characteristics of the two groups. Days from onset to treatment were 6.2 (95% CI: 5.1–7.4) and 4.7 (95% CI: 3.7–5.7) days in the H_2_ and control groups, respectively, which show statistical difference between the two groups (*P* = 0.049). Other parameters such as age, affected side, diabetes, hypertension, hyperlipidemia, vertigo/dizziness, PTA threshold of the affected and contralateral sides, and intratympanic steroid injection as salvage therapy were not significantly different between the two groups.

**TABLE 1:** Baseline patient characteristics.

Characteristics	H_2_ group (*n* = 31)	Control group (*n* = 34)	*P*-value
Age (years, mean ± SE)	60.3 ± 2.8	60.7 ± 2.9	0.91
Days from onset to treatment (95% CI)	6.2 (5.1–7.4)	4.7 (3.7–5.7)	0.049
Affected side (Lt/Rt)	16/15	25/9	0.08
Diabetes (N, %)	6 (19.4%)	13 (38.2%)	0.11
Hypertension (N, %)	13 (41.9%)	17 (50.0%)	0.62
Hyperlipidemia (N, %)	8 (25.8%)	11 (32.4%)	0.60
Vertigo/Dizziness (N, %)	7 (22.6%)	13 (38.2%)	0.19
PTA threshold (dB)			
Affected side (mean, 95% CI)	71.7 (64.8–78.6)	73.7 (65.6–81.8)	0.71
Contralateral side (mean, 95% CI)	23.5 (18.7–28.4)	24.0 (19.8–28.2)	0.88
Intratympanic steroid injection as a salvage therapy (N,%)	7 (22.6%)	11 (32.4%)	0.42

SE, standard error; CI, confidence interval; Lt, left; Rt, right; N, number; PTA, pure tone audiogram; dB, decibels; H_2_, molecular hydrogen.

### The primary outcome

**FIGURE 2:**
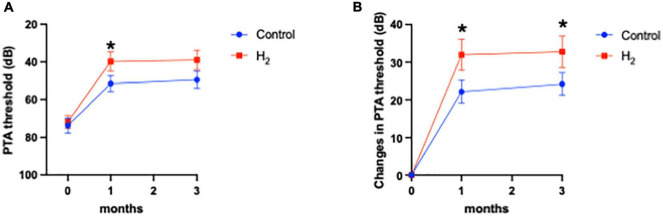
Hearing outcomes. **(A)** PTA threshold. The PTA threshold in the H_2_ group was significantly improved 1 month after treatment. However, this significant difference disappeared 3 months after treatment. *P < 0.05. **(B)** Changes in the PTA threshold. Changes in PTA threshold were significantly greater in the H_2_ group at both 1 and 3 months after treatment. *P < 0.05.

The primary outcome was the hearing threshold and changes in the hearing threshold 3 months after treatment. Notably, the hearing threshold 3 months after treatment was 39.0 dB (95% CI: 28.7–49.3) and 49.5 dB (95% CI: 40.3–58.7) in the H_2_ and control groups, respectively, with no statistical difference between the two groups (*P* = 0.06) (Figure 2A). However, changes in the hearing threshold from the initial visit to 3 months after treatment were 32.7 dB (95% CI: 24.2–41.3) and 24.2 dB (95% CI: 18.1–30.3) in the H_2_ and control groups, respectively, which were significantly better in the H_2_ group than in the control group (*P* = 0.048) (Figure 2B and Supplementary Table 1).

### The secondary outcomes

The hearing threshold 1 month after treatment was 39.8 dB (95% CI: 29.6–49.9) and 51.5 dB (95% CI: 42.9–60.2) in the H_2_ and control groups, respectively. The changes in hearing threshold 1 month after treatment were 31.9 dB (95% CI: 23.5–40.4) and 22.1 dB (95% CI: 15.9–28.4) in the H_2_ and control groups, respectively. Notably, the hearing threshold and changes in hearing threshold 1 month after treatment were significantly better in the H_2_ group (*P* = 0.04 and 0.03, respectively) than those in the control group (Figures 2A,B and Supplementary Table 1).

**FIGURE 3:**
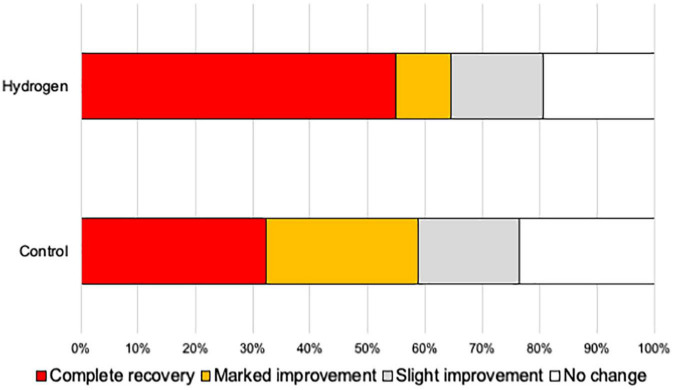
Hearing outcome. The ratio of patients with a good hearing prognosis (complete recovery and marked improvement) was not significantly different between the H_2_ and control groups.

Furthermore, the ratio of good hearing prognosis (complete and partial recoveries) 3 months after treatment was 64.5% (*n* = 20) and 58.8% (*n* = 20) in the H_2_ and control groups, respectively, indicating no significant difference between the two groups (*P* = 0.42) (Figure 3).

**FIGURE 4:**
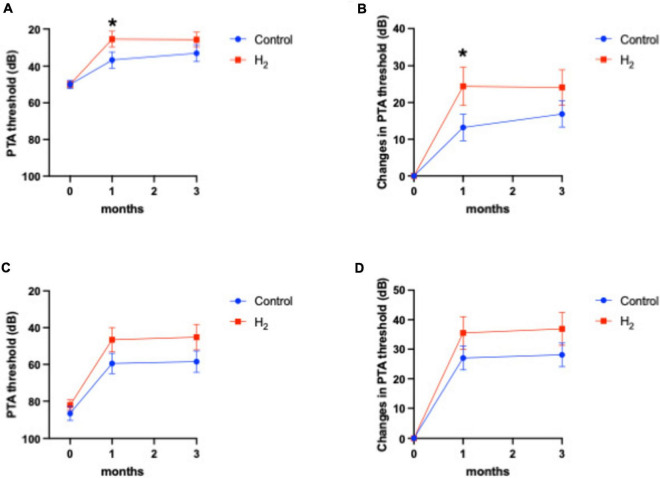
Hearing outcomes depending on the severity of hearing loss at the initial visit. **(A)** PTA threshold among patients with mild hearing loss. **(B)** Changes in the PTA threshold among patients with mild hearing loss. **(C)** PTA threshold among patients with severe hearing loss. **(D)** Changes in the PTA threshold among patients with severe hearing loss. Both the PTA threshold and changes in PTA threshold were significantly better in the H_2_ group 1 month after treatment among patients with mild hearing loss. However, these significant differences disappeared 3 months after treatment. There were no significant differences between the H_2_ and control groups in patients with severe hearing loss. *P < 0.05.

**FIGURE 5:**
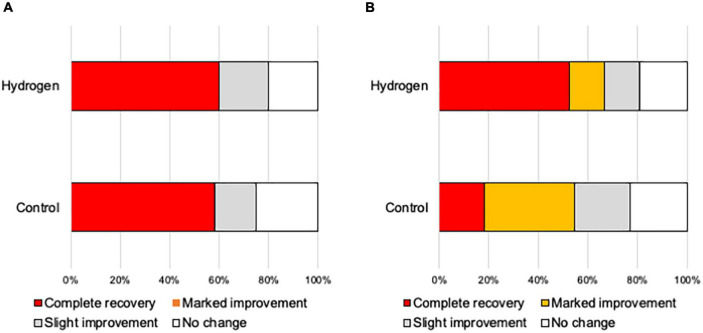
Hearing outcomes depending on the severity of hearing loss at the initial visit. **(A)** Hearing outcomes among patients with mild hearing loss. The ratio of good hearing prognosis (complete recovery and marked improvement) did not vary between the groups. **(B)** Hearing outcomes among patients with severe hearing loss. The ratio of good hearing prognosis did not vary between the groups. However, the ratio of complete recovery was significantly greater in the H_2_ group than in the control group (*P* = 0.02).

Figure 4 shows the hearing outcomes according to the hearing severity at the initial visit (Supplementary Table 2). The mild (PTA < 60 dB) and severe (PTA ≥ 60 dB) hearing loss groups included 22 (10 and 12 in the H_2_ and control groups, respectively) and 43 (21 and 22 in the H_2_ and control groups, respectively) patients, respectively. Among the patients with mild hearing loss at the initial visit, the PTA threshold 1 month after treatment was significantly better in the H_2_ group than in the control group (*P* = 0.04). However, this significant difference disappeared 3 months after treatment (*P* = 0.13) (Figure 4A). In addition, a significant difference was observed between the groups in the changes in hearing threshold 1 month after treatment (*P* = 0.04). However, this difference disappeared 3 months after treatment (*P* = 0.12) (Figure 4B). Furthermore, among the patients with severe hearing loss, there were no significant differences between the groups at the initial visit and 1 and 3 months after treatment (*P* = 0.35, 0.07, and 0.07, respectively) (Figure 4C). The changes in hearing thresholds showed no significant differences between the groups at 1 and 3 months after treatment (*P* = 0.11 and 0.10, respectively) (Figure 4D). Moreover, among the patients with mild hearing loss at the initial visit, the ratio of good hearing prognosis was 60.0% and 58.3% in the H_2_ and control groups, respectively, which shows no significant difference between the groups (*P* = 0.64) (Figure 5A). Among the patients with severe hearing loss at the initial visit, the ratio of good hearing prognosis was 66.7 and 59.1% in the H_2_ and control groups, respectively. Accordingly, there was no significant difference between the groups at the initial visit (*P* = 0.42). However, the ratio of complete recovery was 52.4% and 18.2% in the H_2_ and control groups, respectively, with a significant difference between the groups (*P* = 0.02) (Figure 5B).

**FIGURE 6:**
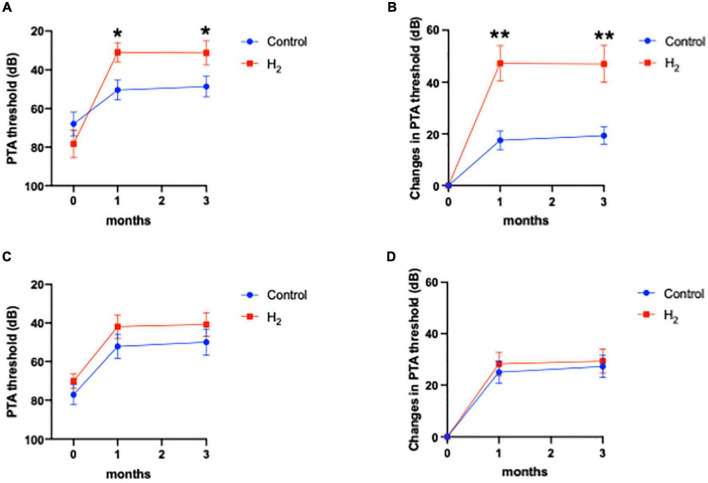
Hearing outcomes depending on diabetes. **(A)** PTA threshold among patients with diabetes. **(B)** Changes in the PTA threshold among patients with diabetes. **(C)** PTA threshold among patients without diabetes. **(D)** Changes in the PTA threshold among patients without diabetes. Among patients with diabetes, both the PTA threshold and changes in PTA threshold were significantly better in the H_2_ group 1 and 3 months after treatment than in the control group. However, these significant differences were not observed among patients without diabetes. *P < 0.05, **P < 0.01.

**FIGURE 7:**
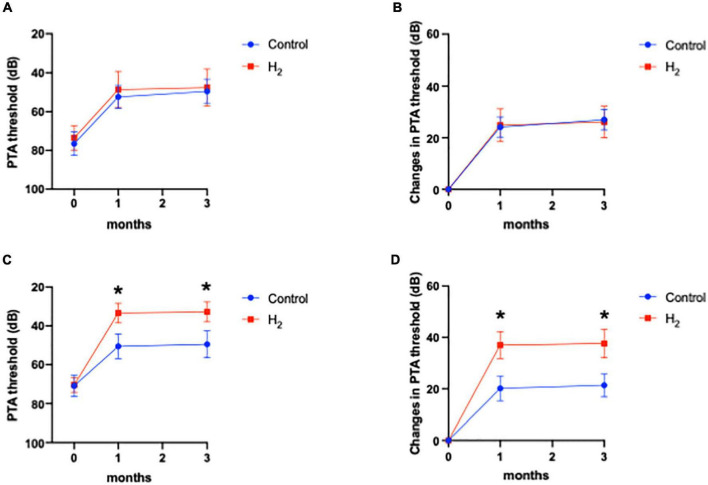
Hearing outcomes depending on hypertension. **(A)** PTA threshold among patients with hypertension. **(B)** Changes in the PTA threshold among patients with hypertension. **(C)** PTA threshold among patients without hypertension. **(D)** Changes in the PTA threshold among patients without hypertension. Although the effect of H_2_ gas inhalation was not observed in patients with hypertension, hearing outcomes were significantly improved in the H_2_ group among patients without hypertension. *P < 0.05.

**FIGURE 8:**
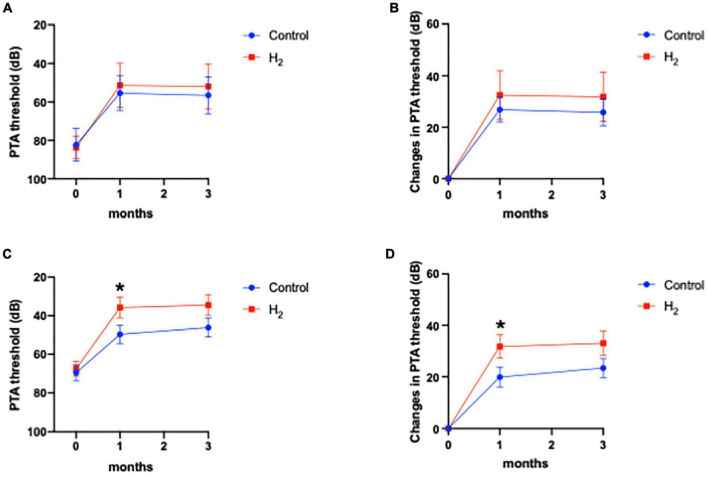
Hearing outcomes depending on hyperlipidemia. **(A)** PTA threshold among patients with hyperlipidemia. **(B)** Changes in the PTA threshold among patients with hyperlipidemia. **(C)** PTA threshold among patients without hyperlipidemia. **(D)** Changes in the PTA threshold among patients without hyperlipidemia. The effect of H_2_ was not observed in patients with hyperlipidemia. Hearing outcomes were significantly better in the H_2_ group 1 month after treatment than in the control group among patients without hyperlipidemia; however, these significant differences disappeared 3 months after treatment. *P < 0.05.

Figure 6 shows that hearing outcomes depend on diabetes (Supplementary Table 3). The PTA threshold was not significantly different at the initial visit among the patients with diabetes. In contrast, the PTA threshold and changes in PTA threshold were significantly better in the H_2_ group than those in the control group among patients with diabetes both at 1 and 3 months after treatment (Figures 6A,B). Notably, among the patients without diabetes, the hearing outcomes were not significantly different between the groups (Figures 6C,D). Figure 6 also shows that hearing outcomes are dependent on hypertension. Among the patients with hypertension, hearing outcomes were not significantly different between the H_2_ and control groups (Figures 7A,B); however, those at 1 and 3 months after treatment were significantly better in the H_2_ group among patients without hypertension (Figures 7C,D and Supplementary Table 4). Figure 8 shows that hearing outcomes depend on hyperlipidemia (Supplementary Table 5). Remarkably, among patients with hyperlipidemia, the hearing outcomes were not significantly different between the groups (Figures 8A,B). In contrast, among patients without hyperlipidemia, the hearing outcomes 1 month after treatment were significantly better in the H_2_ group than in the control group; however, hearing outcomes 3 months after treatment were not significantly different between the groups (Figures 8C,D).

Lastly, side effects due to the inhalation of H_2_ gas were not reported in this study.

### Additional analysis

**FIGURE 9:**
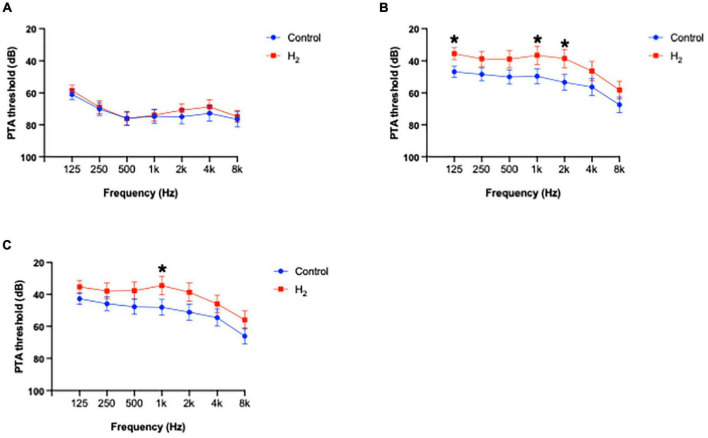
Average of hearing threshold at each frequency. **(A)** PTA threshold at each frequency at initial visit. There were no significant differences between the groups. **(B)** PTA threshold at each frequency 1 month after treatment. The PTA thresholds at 125, 1 k, and 2 k Hz in the H_2_ group were significantly better compared to those in the control group. *P < 0.05. **(C)** PTA threshold at each frequency 3 months after treatment. The PTA threshold 3 months after treatment at 1 k Hz was significantly better in the H_2_ group than in the control group. *P < 0.05.

Figure 9 shows the PTA thresholds at initial visit, 1 and 3 months after treatment at each frequency. The PTA threshold 3 months after treatment at 1 k Hz was significantly better in the H_2_ group than in the control group (*P* = 0.04) (Supplementary Table 6). The changes in PTA thresholds at each frequency are summarized in Supplementary Table 7. The changes in PTA threshold 3 months after treatment were significantly better at 500, 1 k, and 8 k Hz in the H_2_ group than in the control group (*P* = 0.04, 0.02, and 0.03, respectively).

The results of multiple linear regression analysis are summarized in Supplementary Table 8. The changes in PTA threshold at 1 k Hz were significantly associated with treatment (H_2_ or placebo) (*P* = 0.04), suggesting that H_2_ gas inhalation therapy was effective, especially at middle frequencies.

## Discussion

This study proposes that inhalation of H_2_ gas and treatment with systemic glucocorticoids and PGE1 effectively improve hearing outcomes in patients with ISSNHL. To the best of our knowledge, this is the first study to examine the effectiveness of H_2_ gas therapy in ISSNHL treatment.

However, the pathogenesis of ISSNHL remains unknown. Although various causes have been proposed, circulatory disturbance is recognized as the most plausible cause of the disease (Kim et al., 1999). Steroids and vasoactive agents are often used for ISSNHL treatment (Kitoh et al., 2017; Okada et al., 2017). Nevertheless, approximately 40% of patients with ISSNHL have a poor hearing prognosis (Kitoh et al., 2017; Okada et al., 2017). Interestingly, intratympanic steroid (ITS) therapy has recently been used as initial or salvage therapy for ISSNHL; however, the treatment effect of ITS is inadequate (Chandrasekhar et al., 2019).

Inhalation of H_2_ gas was effective in changes in hearing threshold, although the duration from onset to the initial visit was significantly longer in the H_2_ group than in the control group. In animal studies, it has been reported that transient cochlear ischemia causes inner hair cell loss through the depletion of the ATP supply, glutamate excitotoxicity, and cell damage by the generation of free radicals (Hakuba et al., 1997, 2000; Maetani et al., 2003; Morizane et al., 2005). In addition, cochlear blood flow disturbance causes a significant increase in nitric oxide (NO) production in the perilymph, which is attributed to the inducible nitric oxide synthase (iNOS) pathway (Kim et al., 1999). NO production through iNOS is believed to cause injury, which follows ischemia and contributes to neuronal damage through peroxynitrite production (Niwa et al., 1999). Furthermore, our previous study showed that immunostaining for iNOS was strongly expressed on days 1 and 4 after transient cochlear ischemia (Morizane et al., 2005). Notably, H_2_ can selectively scavenge peroxynitrite and hydroxyl radicals produced following transient cochlear ischemia (Ohsawa et al., 2007). In our previous animal study, we also reported that H_2_ is effective for acute hearing loss prevention due to transient cochlear ischemia (Ogawa et al., 2018). Moreover, the therapeutic effects of H_2_ on the inner ear have been reported in animal studies related to noise-induced hearing loss (Lin et al., 2011; Zhou et al., 2012; Chen et al., 2014; Kurioka et al., 2014; Fransson et al., 2021) and ototoxic agents (Qu et al., 2012; Kikkawa et al., 2014; Fransson et al., 2017) through its antioxidative or anti-inflammatory effects (Lin et al., 2011; Qu et al., 2012; Chen et al., 2017). Therefore, H_2_ therapy may be effective for ISSNHL through its antioxidative or anti-inflammatory effects, which include those of the iNOS pathway.

The effect of H_2_ was remarkable in patients with diabetes. Generally, diabetes and hypertension have been reported to contribute to the causes of ISSNHL through microcirculatory disturbances and cellular stress (Teranishi et al., 2007). Both diabetes and hypertension are associated with endothelial dysfunction and oxidative stress (Petrie et al., 2018). Yet, H_2_ was effective only in patients with diabetes whereas ineffective in those with hypertension and hyperlipidemia. Since the mechanisms by which diabetes, hypertension, and hyperlipidemia affect the inner ear pathologies in ISSNHL remain unknown, we cannot explain these results. In addition, further large-scale study in human and animal studies are warranted because of the small number of patients.

Furthermore, the ratio of complete recovery was significantly higher in the H_2_ group among patients with severe hearing loss, although this outcome was not included in the secondary outcomes. Since the severe hearing loss at the initial visit is believed to be a poor prognostic factor, inhalation of H_2_ gas may improve the ISSNHL prognosis. Notably, in this study, there were no adverse effects due to the inhalation of H_2_ gas. In addition, no side effects have been reported in previous studies that administered H_2_ gas to humans (Ishibashi et al., 2014; Katsumata et al., 2017; Ono et al., 2017; Tamura et al., 2020; Botek et al., 2022). This study’s results suggest that the inhalation of H_2_ gas is a safe and effective treatment for severe ISSNHL. However, large-scale studies are required to verify the efficacy of H_2_ gas in treating ISSNHL because of the small number of patients.

This study has several limitations. First, since the number of patients was small, the study population may be insufficient for analysis. In the power analysis of our primary outcome (1-ß), was 0.51, suggesting that large-scale studies are required. In addition, multivariate analysis is more suitable for analyzing the effect of complications on hearing outcomes. However, the number of patients was insufficient for the analysis. Further large-scale studies are needed to confirm the effect of complications. Second, randomization was performed according to the hearing thresholds at the initial visit and age. Although other factors that might influence the hearing outcomes were not significantly different between the H_2_ and control groups, excluding the duration from onset to initial visit, further studies that randomize by including these factors will be required. Third, the optimal concentration of H_2_ and duration of inhalation of H_2_ gas are still unknown for treating ISSNHL. Therefore, further studies are needed to verify these findings. Fourth, we did not perform a speech perception test, as it is not included in the Japanese criteria for hearing outcomes.

This is the first clinical report to analyze the efficacy of H_2_ for ISSNHL. In animal studies, there have been reports that H_2_ alleviates hearing loss due to various causes (Lin et al., 2011; Qu et al., 2012; Zhou et al., 2012; Chen et al., 2014; Kikkawa et al., 2014; Kurioka et al., 2014; Fransson et al., 2017, 2021; Ogawa et al., 2018). However, prior to the present study, no other study had evaluated the efficacy of H_2_ in humans. Further clinical trials to confirm the efficacy of H_2_ in hearing loss by other causes, such as noise-induced or drug-induced, are required. The route of H_2_ administration is roughly classified into three types: inhalation of H_2_ gas, drinking H_2_-dissolved water, and injection of H_2_-dissolved saline (Iketani and Ohsawa, 2017). Inhalation of H_2_ gas is an easy, safe, and reliable way to administer H_2_. No side effects of H_2_ inhalation were reported in this study or in other clinical trials (Ishibashi et al., 2014; Katsumata et al., 2017; Ono et al., 2017; Tamura et al., 2020; Botek et al., 2022). Since there are few disadvantages to inhalation of H_2_ gas, it is expected to be applied to various types of hearing loss.

In conclusion, inhalation of H_2_ gas was effective for treating ISSNHL, and the changes in hearing threshold were significantly better in the H_2_ group than in the control group. The effect of H_2_ gas inhalation was remarkable in patients with diabetes and in those with severe hearing loss. However, H_2_ gas inhalation was ineffective in patients with hypertension but was effective in those without hypertension. Furthermore, no side effects were observed after the inhalation of H_2_ gas. Therefore, these results suggest that the inhalation of H_2_ gas may be safe and effective in ISSNHL treatment.

## Data availability statement

The raw data supporting the conclusions of this article will be made available by the authors, without undue reservation.

## Ethics statement

The studies involving human participants were reviewed and approved by the Certified Review Board of Ehime University. The patients/participants provided their written informed consent to participate in this study.

## Author contributions

MO and NHa designed the study. MO, HO, TT, EN, TY, JH, YS, NHo, MT, and HY collected the data. MO and TF analyzed the data. S-IH prepared the equipment. MO wrote the draft of this manuscript. NHa revised the manuscript. All authors contributed to the manuscript and approved its submission.

## Footnotes

1
https://jrct.niph.go.jp/re/reports/detail/10442

2
http://www.randomizer.org/form.htm

## Abbreviations

ISSNHL: idiopathic sudden sensorineural hearing loss
PGE1: prostaglandin E1
PTA: pure tone audiogram
ITS: intratympanic steroid
ATP: adenosine triphosphate
NO: nitric oxide
iNOS: inducible nitric oxide synthase.
