# Supplementary material for: A double-blinded, randomized controlled clinical trial of hydrogen inhalation therapy for idiopathic sudden sensorineural hearing loss
Source: Front Neurosci. 2022 Nov 24;16:1024634. doi: 10.3389/fnins.2022.1024634 (PMC9731512; doi:10.3389/fnins.2022.1024634)
Supplement: Supplementary file 1 [file Data_Sheet_1.docx]

Supplementary table 1. Hearing threshold and changes in hearing threshold.

| PTA threshold | | | |
| --- | --- | --- | --- |
|  | H_2_ group | Control group | *P*-value |
| Initial visit (dB) | 71.7 (64.8-78.6) | 73.7 (65.6-81.8) | .71 |
| 1 month after treatment (dB) | 39.8 (29.6-49.9) | 51.5 (42.9-60.2) | .04 |
| 3 months after treatment (dB) | 39.0 (28.7-49.3) | 49.5 (40.3-58.7) | .06 |
| Changes in PTA threshold | | | |
|  | H_2_ group | Control group | *P*-value |
| 1 month after treatment (dB) | 31.9 (23.5-40.4) | 22.1 (15.9-28.4) | .03 |
| 3 months after treatment (dB) | 32.7 (24.2-41.3) | 24.2 (18.1-30.3) | .048 |

Data are expressed as mean (95% confidence interval). H_2_, molecular hydrogen; PTA, pure tone audiogram

Supplementary table 2. Hearing outcomes according to the hearing severity at initial visit.

| Among patients with mild hearing loss (PTA < 60 dB) | | | |
| --- | --- | --- | --- |
| PTA threshold | | | |
|  | H_2_ group | Control group | *P*-value |
| Initial visit (dB) | 49.9 (46.2-53.6) | 50.0 (45.2-54.8) | .37 |
| 1 month after treatment (dB) | 25.5 (15.9-35.1) | 36.8 (27.5-46.2) | .04 |
| 3 months after treatment (dB) | 25.8 (16.3-35.3) | 33.1 (24.4-41.7) | .13 |
| Changes in PTA threshold | | | |
|  | H_2_ group | Control group | *P*-value |
| 1 month after treatment (dB) | 24.4 (12.6-36.2) | 13.2 (5.3-21.0) | .04 |
| 3 months after treatment (dB) | 24.1 (13.2-35.0) | 16.9 (8.9-24.9) | .12 |
| Among patients with severe hearing loss (PTA ≥ 60 dB) | | | |
| PTA threshold | | | |
|  | H_2_ group | Control group | *P*-value |
| Initial visit (dB) | 82.1 (76.3-87.9) | 86.6 (78.6-94.5) | .35 |
| 1 month after treatment (dB) | 46.6 (32.8-60.4) | 59.5 (48.1-71.0) | .07 |
| 3 months after treatment (dB) | 45.2 (30.9-59.5) | 58.5 (46.5-70.4) | .07 |
| Changes in PTA threshold | | | |
|  | H_2_ group | Control group | *P*-value |
| 1 month after treatment (dB) | 35.5 (24.1-47.0) | 27.0 (18.8-35.3) | .11 |
| 3 months after treatment (dB) | 36.9 (25.2-48.5) | 28.1 (19.8-36.4) | .10 |

Data are expressed as mean (95% confidence interval). H_2_, molecular hydrogen; PTA, pure tone audiogram

Supplementary table 3. Hearing outcomes depends on diabetes.

| Among patients with diabetes | | | |
| --- | --- | --- | --- |
| PTA threshold | | | |
|  | H_2_ group | Control group | *P*-value |
| Initial visit (dB) | 78.3 (60.2-96.5) | 67.9 (54.6-81.3) | .32 |
| 1 month after treatment (dB) | 31.0 (18.6-43.4) | 50.4 (39.3-61.5) | .02 |
| 3 months after treatment (dB) | 31.3 (15.7-47.0) | 48.6 (37.2-60.1) | .03 |
| Changes in PTA threshold | | | |
|  | H_2_ group | Control group | *P*-value |
| 1 month after treatment (dB) | 47.3 (30.0-64.7) | 17.5 (9.5-25.6) | < .0001 |
| 3 months after treatment (dB) | 47.0 (28.7-65.3) | 19.3 (11.9-26.7) | < .0001 |
| Among patients without diabetes | | | |
| PTA threshold | | | |
|  | H_2_ group | Control group | *P*-value |
| Initial visit (dB) | 70.1 (62.2-78.0) | 77.2 (66.5-88.0) | .13 |
| 1 month after treatment (dB) | 41.9 (29.5-54.3) | 52.2 (39.2-65.2) | .12 |
| 3 months after treatment (dB) | 40.8 (28.3-53.3) | 50.0 (36.1-63.9) | .16 |
| Changes in PTA threshold | | | |
|  | H_2_ group | Control group | *P*-value |
| 1 month after treatment (dB) | 28.2 (18.7-37.8) | 25.0 (15.9-34.1) | .31 |
| 3 months after treatment (dB) | 29.3 (19.6-39.0) | 27.2 (18.2-36.2) | .37 |

Data are expressed as mean (95% confidence interval). H_2_, molecular hydrogen; PTA, pure tone audiogram

Supplementary table 4. Hearing outcomes depends on hypertension.

| Among patients with hypertension | | | |
| --- | --- | --- | --- |
| PTA threshold | | | |
|  | H_2_ group | Control group | *P*-value |
| Initial visit (dB) | 73.5 (59.9-87.2) | 76.5 (63.8-89.1) | .37 |
| 1 month after treatment (dB) | 48.6 (28.3-68.9) | 52.4 (39.9-64.9) | .36 |
| 3 months after treatment (dB) | 47.5 (26.7-68.3) | 49.5 (36.5-62.4) | .43 |
| Changes in PTA threshold | | | |
|  | H_2_ group | Control group | *P*-value |
| 1 month after treatment (dB) | 24.9 (10.9-38.9) | 24.1 (15.7-32.4) | .45 |
| 3 months after treatment (dB) | 26.1 (12.8-39.3) | 27.0 (18.5-35.5) | .55 |
| Among patients without hypertension | | | |
| PTA threshold | | | |
|  | H_2_ group | Control group | *P*-value |
| Initial visit (dB) | 70.4 (62.4-78.4) | 70.9 (59.5-82.3) | .47 |
| 1 month after treatment (dB) | 33.4 (22.8-44.0) | 50.6 (37.2-64.1) | .02 |
| 3 months after treatment (dB) | 32.8 (22.1-43.6) | 49.5 (34.9-64.1) | .03 |
| Changes in PTA threshold | | | |
|  | H_2_ group | Control group | *P*-value |
| 1 month after treatment (dB) | 37.0 (25.9-48.1) | 20.2 (10.1-30.4) | .01 |
| 3 months after treatment (dB) | 37.6 (25.8-49.3) | 21.4 (11.9-30.8) | .02 |

Data are expressed as mean (95% confidence interval). H_2_, molecular hydrogen; PTA, pure tone audiogram

Supplementary table 5. Hearing outcomes depends on hyperlipidemia.

| Among patients with hyperlipidemia | | | |
| --- | --- | --- | --- |
| PTA threshold | | | |
|  | H_2_ group | Control group | *P*-value |
| Initial visit (dB) | 83.8 (69.8-97.7) | 82.3 (63.3-101.3) | .45 |
| 1 month after treatment (dB) | 51.3 (24.2-78.3) | 55.4 (35.3-75.5) | .39 |
| 3 months after treatment (dB) | 51.9 (24.2-79.5) | 56.5 (35.1-77.8) | .39 |
| Changes in PTA threshold | | | |
|  | H_2_ group | Control group | *P*-value |
| 1 month after treatment (dB) | 32.5 (10.0-55.0) | 26.9 (16.3-37.5) | .29 |
| 3 months after treatment (dB) | 31.9 (9.4-54.4) | 25.8 (14.2-37.5) | .28 |
| Among patients without hyperlipidemia | | | |
| PTA threshold | | | |
|  | H_2_ group | Control group | *P*-value |
| Initial visit (dB) | 67.5 (59.8-75.3) | 69.6 (61.0-78.1) | .36 |
| 1 month after treatment (dB) | 35.8 (24.8-46.8) | 49.7 (39.9-59.4) | .03 |
| 3 months after treatment (dB) | 34.5 (23.5-45.4) | 46.2 (36.0-56.3) | .06 |
| Changes in PTA threshold | | | |
|  | H_2_ group | Control group | *P*-value |
| 1 month after treatment (dB) | 31.7 (22.1-41.4) | 19.9 (11.8-27.9) | .03 |
| 3 months after treatment (dB) | 33.0 (23.3-42.8) | 23.4 (15.6-31.1) | .06 |

Data are expressed as mean (95% confidence interval). H_2_, molecular hydrogen; PTA, pure tone audiogram

Supplementary table 6. Hearing thresholds in each frequency.

| PTA threshold at initial visit | | | |
| --- | --- | --- | --- |
|  | H_2_ group | Control group | *P*-value |
| 125 Hz (dB) | 58.5 (52.1-65.0) | 61.0 (54.8-67.2) | .58 |
| 250 Hz (dB) | 69.0 (60.9-77.2) | 70.1 (62.4-77.9) | .84 |
| 500 Hz (dB) | 76.1 (67.7-84.5) | 75.9 (67.9-83.9) | .97 |
| 1k Hz (dB) | 73.9 (65.6-82.1) | 74.7 (66.8-82.6) | .88 |
| 2k Hz (dB) | 70.8 (62.2-79.4) | 74.9 (66.6-83.1) | .50 |
| 4k Hz (dB) | 68.7 (59.2-78.2) | 72.8 (63.7-81.9) | .54 |
| 8k Hz (dB) | 74.8 (66.1-83.6) | 76.6 (68.3-85.0) | .77 |
| PTA threshold 1 month after treatment | | | |
|  | H_2_ group | Control group | *P*-value |
| 125 Hz (dB) | 35.5 (28.1-42.9) | 46.8 (39.7-54.0) | .02 |
| 250 Hz (dB) | 38.7 (29.8-47.6) | 48.4 (39.9-56.9) | .06 |
| 500 Hz (dB) | 38.9 (29.0-48.8) | 50.0 (40.6-59.4) | .05 |
| 1k Hz (dB) | 36.5 (26.0-46.9) | 49.6 (39.6-59.5) | .04 |
| 2k Hz (dB) | 38.5 (27.8-49.3) | 53.4 (43.1-63.6) | .03 |
| 4k Hz (dB) | 46.3 (35.1-57.4) | 56.3 (45.7-67.0) | .10 |
| 8k Hz (dB) | 58.2 (47.8-68.7) | 67.4 (57.4-77.3) | .11 |
| PTA threshold 3 months after treatment | | | |
|  | H_2_ group | Control group | *P*-value |
| 125 Hz (dB) | 35.3 (27.8-42.8) | 42.8 (35.6-50.0) | .08 |
| 250 Hz (dB) | 37.9 (28.8-47.0) | 45.9 (37.2-54.5) | .10 |
| 500 Hz (dB) | 37.7 (27.5-48.0) | 47.8 (38.0-57.6) | .08 |
| 1k Hz (dB) | 34.5 (23.6-45.4) | 48.1 (37.7-58.5) | .04 |
| 2k Hz (dB) | 38.7 (27.8-49.6) | 51.2 (40.7-61.6) | .05 |
| 4k Hz (dB) | 46.0 (35.0-57.0) | 54.6 (44.1-65.1) | .13 |
| 8k Hz (dB) | 56.0 (45.2-66.8) | 66.2 (55.9-76.5) | .09 |

Data are expressed as mean (95% confidence interval). H_2_, molecular hydrogen; PTA, pure tone audiogram

Supplementary table 7. Changes in hearing thresholds in each frequency.

| Changes in PTA threshold 1 month after treatment | | | |
| --- | --- | --- | --- |
|  | H_2_ group | Control group | *P*-value |
| 125 Hz (dB) | 23.1 (15.8-30.3) | 15.6 (8.7-22.5) | .07 |
| 250 Hz (dB) | 30.3 (22.0-38.7) | 21.8 (13.8-29.7) | .07 |
| 500 Hz (dB) | 37.3 (28.6-45.9) | 25.9 (17.6-34.2) | .03 |
| 1k Hz (dB) | 37.4 (28.9-45.9) | 25.1 (17.0-33.3) | .02 |
| 2k Hz (dB) | 32.3 (24.2-40.3) | 21.5 (13.7-29.2) | .03 |
| 4k Hz (dB) | 22.4 (15.3-29.6) | 16.5 (9.6-23.3) | .12 |
| 8k Hz (dB) | 16.6 (10.7-22.5) | 9.3 (3.6-14.9) | .04 |
| PTA threshold 3 months after treatment | | | |
|  | H_2_ group | Control group | *P*-value |
| 125 Hz (dB) | 23.2 (15.7-30.7) | 18.2 (11.1-25.4) | .17 |
| 250 Hz (dB) | 31.1 (22.8-39.5) | 24.3 (16.3-32.3) | .12 |
| 500 Hz (dB) | 38.4 (29.9-46.8) | 28.1 (20.0-36.2) | .04 |
| 1k Hz (dB) | 39.4 (30.7-48.0) | 26.6 (18.4-34.8) | .02 |
| 2k Hz (dB) | 32.1 (23.9-40.3) | 23.7 (15.8-31.5) | .07 |
| 4k Hz (dB) | 22.7 (15.7-29.8) | 18.2 (11.5-24.9) | .18 |
| 8k Hz (dB) | 18.9 (12.6-25.2) | 10.4 (4.4-16.5) | .03 |

Data are expressed as mean (95% confidence interval). H_2_, molecular hydrogen; PTA, pure tone audiogram

Supplementary table 8. Analysis of multivariable linear model in changes in PTA thresholds.

| 1 month after treatment | | | | |
| --- | --- | --- | --- | --- |
| Frequency | Variable | ß | SD | *P*-value |
| 125 Hz | Treatment | -3.71 | 2.52 | .15 |
|  | Age | -0.11 | 0.16 | .49 |
| 250 Hz | Treatment | -4.26 | 2.9 | .15 |
|  | Age | -0.09 | 0.18 | .61 |
| 500 Hz | Treatment | -5.68 | 3.02 | .06 |
|  | Age | -0.04 | 0.19 | .82 |
| 1k Hz | Treatment | -6.08 | 2.92 | .04 |
|  | Age | -0.22 | 0.18 | .22 |
| 2k Hz | Treatment | -5.35 | 2.79 | .06 |
|  | Age | -0.2 | 0.17 | .24 |
| 4k Hz | Treatment | -2.93 | 2.47 | .24 |
|  | Age | -0.18 | 0.15 | .25 |
| 8k Hz | Treatment | -3.61 | 1.99 | .07 |
|  | Age | -0.26 | 0.12 | .04 |
| 3 months after treatment | | | | |
| Frequency | Variable | ß | SD | *P*-value |
| 125 Hz | Treatment | -2.47 | 2.61 | .35 |
|  | Age | -9 | 0.16 | .59 |
| 250 Hz | Treatment | -3.43 | 2.92 | .24 |
|  | Age | 0.00 | 0.18 | .99 |
| 500 Hz | Treatment | -5.17 | 2.95 | .08 |
|  | Age | 0.07 | 0.18 | .71 |
| 1k Hz | Treatment | -6.33 | 3 | .04 |
|  | Age | -0.13 | 0.18 | .48 |
| 2k Hz | Treatment | -4.17 | 2.85 | .15 |
|  | Age | -0.15 | 0.18 | .40 |
| 4k Hz | Treatment | -2.21 | 2.42 | .36 |
|  | Age | -0.18 | 0.15 | .23 |
| 8k Hz | Treatment | -4.17 | 2.16 | .06 |
|  | Age | -0.21 | 0.13 | .13 |
